# Frequency-Specific Changes of Amplitude of Low-Frequency Fluctuations in Patients with Acute Basal Ganglia Ischemic Stroke

**DOI:** 10.1155/2022/4106131

**Published:** 2022-01-24

**Authors:** Xuemei Quan, Su Hu, Chaoguo Meng, Lulu Cheng, Yujie Lu, Yumei Xia, Wenmei Li, Huo Liang, Mengting Li, Zhijian Liang

**Affiliations:** ^1^Department of Neurology, The First Affiliated Hospital of Guangxi Medical University, Nanning, China; ^2^Department of Neurology, The People's Hospital of Guangxi Zhuang Autonomous Region, Nanning, China; ^3^School of Teacher Education, Zhejiang Normal University, Jinhua, China; ^4^Key Laboratory of intelligent Education Technology and Application of Zhejiang Province, Zhejiang Normal University, Jinhua, China; ^5^School of Foreign Studies, China University of Petroleum, Qingdao, China; ^6^Shanghai Center for Research in English Language Education, Shanghai International Studies University, Shanghai, China; ^7^Department of Radiology, The First Affiliated Hospital of Guangxi Medical University, Nanning, China; ^8^Department of Radiology, Sichuan Cancer Hospital and Institute, Sichuan Cancer Center, School of Medicine, University of Electronic Science and Technology of China, Chengdu, China; ^9^Department of Rehabilitation, Guangxi International Zhuang Medicine Hospital, No. 8 Qiuyue Road, Nanning, China

## Abstract

**Objective:**

The purpose of this study was to investigate the characteristics of different frequency bands in the spontaneous brain activity among patients with acute basal ganglia ischemic stroke (BGIS).

**Methods:**

In the present study, thirty-four patients with acute BGIS and forty-four healthy controls were examined by resting-state functional magnetic resonance imaging (rs-fMRI) from May 2019 to December 2020. Two amplitude methods including amplitude of low-frequency fluctuations (ALFF) and fractional ALFF (fALFF) calculated in three frequency bands (conventional frequency band: 0.01-0.08 Hz; slow-5 frequency band: 0.01-0.027 Hz; and slow-4 frequency band: 0.027-0.073 Hz) were conducted to evaluate the spontaneous brain activity in patients with acute BGIS and healthy controls (HCs). Gaussian Random Field Theory (GRF, voxel *p* < 0.01 and cluster *p* < 0.05) correction was applied. The correlation analyses were performed between clinical scores and altered metrics values.

**Results:**

Compared to HCs, patients with acute BGIS showed decreased ALFF in the right supramarginal gyrus (SMG) in the conventional and slow-4 bands, increased fALFF in the right middle frontal gyrus (MFG) in the conventional and slow-4 bands, and increased fALFF in the bilateral caudate in the slow-5 frequency band. The fALFF value of the right caudate in the slow-5 frequency band was negatively correlated with the clinical scores.

**Conclusion:**

In conclusion, this study showed the alterations in ALFF and fALFF in three frequency bands between patients with acute BGIS and HCs. The results reflected that the abnormal LFO amplitude might be related with different frequency bands and promoted our understanding of pathophysiological mechanism in acute BGIS.

## 1. Introduction

Acute stroke is a prominent cause of mortality and disability worldwide and leads to a heavy burden on individual, families, and society [1–3]. Acute ischemic stroke which is caused by a sudden interruption of blood supply resulted from to occlusion or obstruction by a thrombus or embolus accounts for more than 80% of all strokes [4]. The basal ganglia region is rich in blood supply and is a common site of ischemic stroke [5]. Acute ischemic stroke occurring in the basal ganglia region is defined as acute basal ganglia ischemic stroke (BGIS). The acute BGIS has been considered to have association with motor impairments, sensory disturbance, emotional blunting, poststroke depression, and loss of spontaneous speech [6–8]. Therefore, the exploration of the pathophysiological mechanism of acute BGIS is vital and could provide potential help for the rehabilitation therapy in the patients with acute BGIS.

Resting-state functional magnetic resonance imaging (rs-fMRI) was first proposed by Biswal et al. to explore brain activity in 1995 [9]. As a noninvasive method to study brain physiological activity, it has been widely used in neuropsychiatric diseases [10–12]. Previous studies have found the disrupted functional connectivity (FC) in acute BGIS patients which characterized the function of whole-brain network [13, 14]. However, the local variation in brain function of acute BGIS patients remains to be explored. At present, various methods, such as amplitude of low frequency fluctuation (ALFF) and fractional ALFF (fALFF), can be used to analyze the changes of brain function [9, 15, 16]. As a promising method, the ALFF is proposed to examine the blood oxygen level-dependent (BOLD) signal fluctuations and to measure the local brain activity of diseases [15–17]. Based on ALFF, Zou et al. proposed the fALFF which is the ratio of the power spectrum of low frequency to that of the entire frequency range [16]. Unlike ALFF, which indicates the strength and intensity of the low-frequency oscillations (LFOs), fALFF reflects the relative contribution of specific LFOs to the whole detectable frequency range [18]. Evidence shows that fALFF can inhibit the nonspecific signal components in fMRI, making the detection of spontaneous brain activities more sensitive and specific [16], while ALFF often has higher reliability than fALFF in the test-retest reliability of amplitude measures [18]. Hence, the combination of these two methods mentioned above can reflect a more complete picture of spontaneous neural activity of the brain. Also, these two methods have been widely used in rs-fMRI studies of neurocognitive diseases [11, 19, 20].

Most studies using the two methods focused on the LFOs which is known as conventional frequency band ranging from 0.01 to 0.08 Hz. The human brain is capable of executing complex functions which are supported by a multitude of oscillatory waves [18]. Based on natural logarithmic linear theory of neural oscillations, the neural oscillations of the human brain can be divided into different frequency bands, and the neural oscillations of different frequency bands show different brain functions [21]. The conventional frequency band is related to the spontaneous neural activities and has the physiological meaning [19]. However, a recent study has found that the results obtained from conventional frequency band only are lack of frequency characteristics [22]. Zuo et al. [18] found that the conventional frequency band of 0.01-0.08 Hz could be divided into two subfrequency bands: slow-4 frequency band of 0.027-0.073 Hz and slow-5 frequency band of 0.01-0.027 Hz. Slow-4 band (0.027-0.073 Hz) mainly reflects the alteration of gray matter signals, while slow-5 band (0.01-0.027 Hz) could reflect the alteration of ventromedial prefrontal cortices [18]. LFOs at different frequency bands show different properties and physiological functions [18, 21, 23]. Meanwhile, the spontaneous LFOs of the subcortical stroke are thought to contain frequency-dependent rs-fMRI patterns, which may serve as potential neuroimaging markers of the neural substrates associated with hand function outcomes following stroke [24]. However, the characteristics of different frequency bands to the spontaneous brain activity in patients with acute BGIS remain unclear. The mechanism of low frequency oscillation in BGIS in specific frequency band needs to be further explored.

In the present study, ALFF and fALFF were used to demonstrate the frequency-specific alteration of the local spontaneous brain activity of acute BGIS patients. The correlations between LFOs in significant brain regions and clinical scores were explored in the patient group.

## 2. Materials and Methods

### 2.1. Participants

From May 2019 to December 2020, 43 acute BGIS patients who had neurologic symptoms and have been judged by clinical neurologists were consecutively recruited from Department of Neurology, the First Affiliated Hospital of Guangxi Medical University. The inclusion criteria for all patients are as follows: (1) first onset acute BGIS diagnosed by a consensus of a clinical neurologist and a radiologist; (2) age between 30 and 75 years; (3) illness duration of stroke less than 10 days; (4) right-handedness before stroke; (5) the National Institutes of Health Stroke Scale (NIHSS) scores ranging from 0 to 16. The exclusion criteria of this study included the following: (1) inability to perform clinical scale examination, such as severe aphasia, auditory, and/or visual disorder; (2) other neurological disorders that would affect the experiment, such as hemorrhage, multiple infarcts, leukoaraiosis, migraine, epilepsy, or psychiatric diseases; (3) any contraindications for MRI, including pregnancy and metal implants; and (4) excessive head motion during rs-fMRI scanning. This protocol was approved by the Ethics Committee of the First Affiliated Hospital of Guangxi Medical University. All participants signed a written informed consent before the study.

In this study, forty-seven age-matched healthy controls (HCs) with no physical diseases or history of psychiatric or neurologic disorders who were recruited from local community were also recruited through advertising at the same time.

Nine patients were excluded from the final analysis due to poor image quality (*n* = 1), excessive head motion (*n* = 2), missing data (*n* = 1), and incomplete scanning of cerebellum (*n* = 5), leaving 34 acute BGIS patients in the final analysis. Three HCs were excluded from the final analysis due to poor image quality (*n* = 1), excessive head motion (*n* = 1), and incomplete scanning of cerebellum (*n* = 1), leaving 44 HCs in the final analysis.

### 2.2. Clinical Scale Tests

Stroke severity and neurological deficits were assessed using the NIHSS. And motor function was assessed by the Fugl-Meyer Assessment (FMA) scale.

### 2.3. MRI Data Acquisition

For each participant, a total of 186 time points (6 minutes and 12 seconds) were collected on a 3.0 T MR scanner (SIEMENS MAGNETOM Prisma), which is equipped with a 64-channel phased array head coil at 3.0 T MRI center, in the First Affiliated Hospital of Guangxi Medical University. During the data acquisition, participants were instructed to keep awake, relax with their eyes closed, and remain motionless as much as possible.

The rs-fMRI was acquired using an echoplanar imaging (EPI) sequence with the following parameters: repetition time (TR) = 2000 ms, echo time (TE) = 35 ms, flip angle (FA) = 90°, field of view (FOV) = 240 × 240 mm^2^, voxel size = 2.6 × 2.6 × 3 mm^3^, matrix = 64 × 64, gap = 0 mm, and slice number = 40. This session lasted for 6 minutes and 12 seconds.

The anatomical 3D-MPRAG T1-weighted images (T1WI) were recorded by magnetization prepared rapid gradient echo: TR = 2300 ms, TE = 2.98 ms, reverse time = 900 ms, FOV = 256 × 256 mm^2^, voxel size = 1 × 1 × 1 mm^3^, matrix = 256 × 256, gap = 0 mm, and slice number = 176. This session lasted for 5 minutes and 21 seconds.

### 2.4. RS-fMRI Data Preprocessing

All algorithms were implemented in Matlab R2018a (https://uk.mathworks.com/products/matlab). RS-fMRI data preprocessing and statistical analyses were carried out using RESTplus V1.24 (http://www.restfmri.net), and SPM12 (http://www.fil.ion.ucl.ac.uk/spm/software/spm12/); multiple comparison corrections were performed using the Data Processing & Analysis for Brain Imaging (DPABI) V5.1 (http://rfmri.org/dpabi). The preprocessing steps included the following: (1) removing the first 10 time points to make the longitudinal magnetization achieve steady-state and to let the participants get used to the scanning environment; (2) slice timing correction; (3) head motion correction. We excluded the participants whose head motion exceeded 3 mm or 3°. Hence, two patients and one HC were excluded; (4) the functional images were spatially normalized to the Montreal Neurological Institute (MNI) space via the deformation fields derived from new segmentation of structural images (resampling voxel size = 3 mm × 3 mm × 3 mm); (5) spatial smoothing with a Gaussian kernel of 4 mm full-width at half-maximum (FWHM); (6) removing the linear trend of the time series; (7) regressing out nuisance variables, including the Friston-24 head motion parameters [25], polynomial trend, white matter signals, and cerebrospinal flow signals.

### 2.5. ALFF Calculation

The ALFF is a rs-fMRI metric that calculates the amplitude of each voxel in the local brain region in conventional frequency band of 0.01-0.08 Hz and reflects the extent of local spontaneous neuronal activity in the resting state [15, 26]. For ALFF calculations, the time series of each voxel was transformed to frequency domain by fast Fourier transform (FFT) and the power spectrum was then obtained. The square root was calculated at each frequency of the power spectrum, and the averaged square root obtained across conventional frequency band at each voxel was regarded as the ALFF value, which was further divided by the global mean ALFF of each participant. In order to study the frequency-dependent changes in BGIS patients during the acute phase, we also calculated the ALFF values of subfrequency bands including slow-4 frequency band of 0.027-0.073 Hz and slow-5 frequency band of 0.01-0.027 Hz.

### 2.6. fALFF Calculation

After data preprocessing, the BOLD signal was converted from time domain to frequency domain by FFT formula, and the power spectrum of BOLD signal in frequency domain was obtained. The power spectrum of BOLD signal in frequency domain was obtained by square calculation. The ratios of the power in the conventional frequency band were calculated relative to the full frequency band (0-0.25 Hz). The result mentioned above was known as fALFF. Then, fALFF divided by global mean fALFF of every individual was applied for standardization purposes. To investigate the frequency-dependent alterations in BGIS patients during the acute phase, we also calculated the fALFF of sub-frequency bands including slow-4 frequency band (0.027-0.073 Hz) and slow-5 frequency band (0.01-0.027 Hz).

### 2.7. Statistical Analysis

Statistical analyses were performed using SPSS version 26.0 (IBM, Armonk, NY, USA). Categorical variables are presented as *n*, and continuous variables are presented as the mean ± standard deviation (SD). Gender difference was tested with a chi-square test. A two-sample *t*-test was performed to compare the age difference between the acute BGIS patients and HCs. All tests of demographic were two-tailed, and *p* < 0.05 was considered significant.

Two sample *t*-tests were performed to compare the ALFF and fALFF maps between patients with BGIS and HCs, respectively. Frame-wise displacement (FD, Jenkinson [27]) parameters were regressed in the two-sample *t*-test to avoid the influence of head motion. The resultant T-maps were conducted with Gaussian Random Field Theory (GRF) correction for multiple comparisons with voxel *p* < 0.01, cluster *p* < 0.05.

For each metric (ALFF, fALFF) which shows acute BGIS related alterations, Pearson's correlation analysis was used to assess their associations with clinical scales (NIHSS scores and FMA scores) of patients. The correlations were considered significant at a threshold of *p* < 0.05.

## 3. Results

### 3.1. Participants' Characteristics

**Figure 1:**
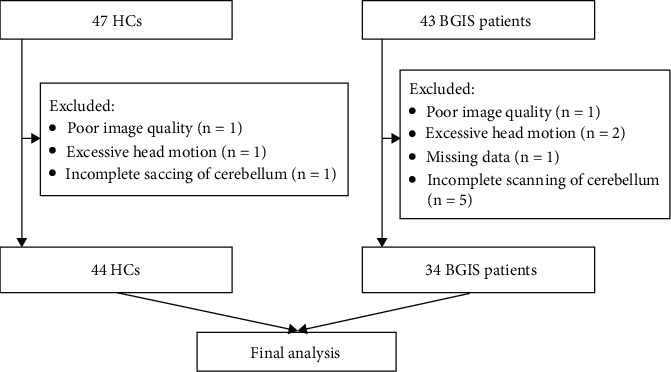
Flow chart. HCs: healthy controls; BGIS: basal ganglia ischemic stroke.

A total of 43 patients with acute BGIS and 47 HCs underwent the rs-fMRI scan in the present study from May 2019 to December 2020. Nine patients and three HCs were excluded according to the above exclusion criteria (Figure 1). Demographics and clinical data of the acute BGIS patients and HCs were calculated (Table 1). There were no significant differences in age (*p* = 0.736) but in gender (*p* = 0.007) between acute BGIS patients and HCs. The statistical analysis results after gender regressed out are provided in the supplementary materials (Table S1, Figure S1 and Figure S2 in the supplementary materials). The lesion map for enrolled acute basal ganglia ischemic stroke is displayed in the supplementary materials (Figure S3 in the supplementary materials).

### 3.2. Disrupted Local Function in BGIS in Multifrequency Bands

#### 3.2.1. ALFF Analysis in Different Frequency Bands

**Figure 2:**
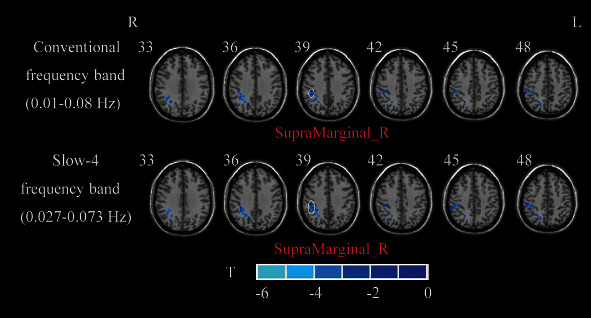
A two-sample *t*-test was performed between acute BGIS patients and HCs. R: right hemisphere; L: left hemisphere; BGIS: basal ganglia ischemic stroke; HCs: healthy controls; ALFF: amplitude of low frequency fluctuation.

Compared with HCs, the BGIS patients exhibited decreased ALFF in the right supramarginal gyrus (SMG) in conventional frequency band and slow-4 frequency band (GRF correction, voxel *p* < 0.01, cluster *p* < 0.05). No significant brain region was found between acute BGIS patients and HCs in slow-5 band (Table 2 and Figure 2).

#### 3.2.2. fALFF Analysis in Different Frequency Bands

**Figure 3:**
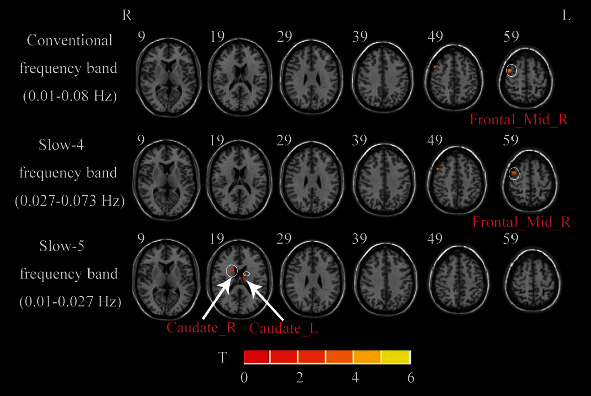
A two-sample *t*-test was performed between acute BGIS patients and HCs. R: right hemisphere; L: left hemisphere; BGIS: basal ganglia ischemic stroke; HCs: healthy controls; fALFF: fractional amplitude of low frequency fluctuation.

Compared with HCs, the BGIS patients exhibited increased fALFF in the right middle frontal gyrus (MFG) in conventional frequency band and slow-4 frequency band (GRF correction, voxel *p* < 0.01, cluster *p* < 0.05). The cluster size of the right MFG in the conventional frequency band was larger than that of slow-4 band (Table 2 and Figure 3).

Compared with HCs, the BGIS patients exhibited increased fALFF in the bilateral caudate in the slow-5 frequency band (GRF correction, voxel *p* < 0.01, cluster *p* < 0.05) (Table 2 and Figure 3).

### 3.3. Relationship Between Local Metrics and Clinical Scales

**Figure 4:**
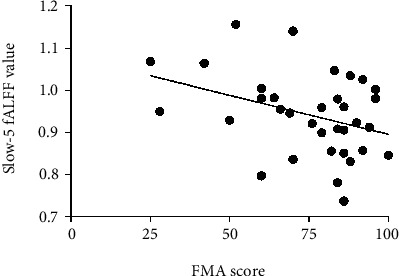
Abnormal cluster of the fALFF was significantly correlated with FMA score in BGIS patients in the slow-5 band (*p* < 0.05). BGIS: basal ganglia ischemic stroke; fALFF: fractional amplitude of low frequency fluctuation; FMA: Fugl-Meyer Assessment scale.

In the BGIS patients, increased fALFF values of the slow-5 frequency band in the right caudate were negatively correlated with FMA scores (*p* = 0.035, *r* = −0.363) (Table 3 and Figure 4). However, no significant correlations were detected between abnormal ALFF values in the conventional frequency band and slow-4 band and clinical scores (Table 3 and Figure 4).

## 4. Discussion

In the present study, we utilized ALFF and fALFF to investigate the sensitivity and characteristics of the LFOs of the spontaneous neural activity in the conventional frequency band (0.01-0.08 Hz), slow-5 frequency band (0.01-0.027 Hz), and slow-4 frequency band (0.027-0.073 Hz) in acute BGIS patients and further explored the relationship between metrics values and the clinical scales. Compared to the HCs, the acute BGIS patients exhibited decreased ALFF values in the right SMG in the conventional band and slow-4 band and increased fALFF in the right MFG in the conventional band and slow-4 band and bilateral caudate in the slow-5 band. In the ALFF analysis of the three frequency bands, the clusters detected in the slow-4 band were larger than those in the conventional frequency band, suggesting that the slow-4 band was more sensitive to the detection of spontaneous brain activity in the ALFF in patients with acute BGIS. However, increased fALFF in the bilateral caudate were only observed in the slow-5 band. In addition, increased fALFF values in the right caudate in the slow-5 band were negatively correlated with FMA scores. These findings indicated that frequency-dependent alterations in intrinsic activity of specific brain regions may serve as potential neuroimaging markers for the mechanism of pathophysiology in patients with acute BGIS.

ALFF was thought to reflect the degree of spontaneous neuronal activity [15]. The increased ALFF indicates the increase of neuronal excitability and metabolism, while the decreased ALFF indicates the inhibition of neuronal spontaneous activity. In the present study, the right SMG of BGIS patients showed decreased ALFF, which indicated decreased spontaneous neuronal activity in this brain region compared with HCs. The SMG was involved in verbal working memory, phonological processing/storage of speech (i.e., spoken and written language), and motor control [28–30]. A previous study showed that decreased functional connectivity (FC) between hippocampal and SMG was associated with impaired working memory function in stroke patients [31]. Similar results were also found in the previous studies focusing on the patients with acute BGIS which observed the decreased degree centrality (DC) and voxel-mirrored homotopic connectivity (VMHC) values in SMG [32]. Furthermore, aphasia is a common symptom in stroke [33]. And the brain injury in SMG has been proposed to be associated with poststroke aphasia [34]. Also, one study has revealed the association between phonological agraphia and SMG in patients with ischemic stroke [35]. In addition to the studies focusing on motor dysfunction, numerous functional neuroimaging studies showed that stroke survivors exhibited deficits of learning, memory, language processing function, and cognition [36–39]. The results of the present study supported the findings above from the perspective of neuroimaging.

fALFF reflects the ratio of power of low-frequency band to that of detectable frequency range [16]. fALFF has been described as “superior” in comparisons to ALFF due to its higher specificity in capturing gray matter signal [16, 40]. The MFG, located in Brodmann areas 9 and 10, constitutes an important part of the cognitive function [41]. In the present study, several brain regions with different fALFF values in the right MFG were identified, which echoes the finding of previous studies [42, 43]. Moreover, the fALFF value changes from increased to decreased in MFG after repetitive transcranial magnetic stimulation (rTMS) in the patient with poststroke cognitive impairment [42]. The observed increased fALFF value in the MFG in the current study further verified the importance of the MFG in acute BGIS patients and enriched the pathogenesis research of acute BGIS.

The acute BGIS patients exhibited increased fALFF value in the bilateral caudate in the present study which showed the functional compensation in the caudate. A previous study has revealed that the bilateral caudate regions are involved in the impaired connections in the early-state stroke patients [44]. In addition, the caudate is associated with language production such as monitoring and controlling lexical and language alternatives [45]. The abnormal connections in the poststroke patients may led to poor language task performance. Meanwhile, the involvement in motor control of caudate has also been found [46]. The research of Nierhaus and colleagues has showed increased functional connectivity between the caudate nucleus and red nucleus in patients with poststroke motor disturbance [47]. Li et al. who used graph-based theoretical approach found that the nodal efficiency of acute stroke patients with unimanual motor deficits was reduced in the caudate [48]. However, correlation results in the current study showed that there was a significant negative correlation between FMA scores and fALFF value of the right caudate. The higher the spontaneous nerve activity of caudate, the more obvious the motor deficits. There is an imbalance between spontaneous activity in right caudate and clinical manifestations. It is speculated that in the process of acute BGIS, right caudate attempted to maintain motor function by increasing the spontaneous activity. However, compensation is difficult to maintain and gradually enters the stage of decompensation as the progress of pathology.

In the current study, the ALFF in the acute BGIS patients decreased in the SMG in the slow-4 frequency band but there was no significant alteration in the slow-5 frequency band. It indicated that the spontaneous activity was sensitive to specific frequency bands. The slow-4 band might be more sensitive to detect abnormal intrinsic brain activity in the SMG in patients with acute BGIS. The similar pattern was also observed in other disorders. Zhang et al. [49] revealed the sensitivity of slow-4 band in detecting the intrinsic activity in Parkinson's disease (PD). A study using ReHo method also reported that ReHo changed significantly in the slow-4 band rather than in the slow-5 band [50].

As for the results of fALFF, increased fALFF was found in conventional band and slow-4 band. However, the increased fALFF in the bilateral caudate was only detected in the slow-5 frequency band. It reflected that the results in the conventional band were mainly contributed by results in the slow-4 band, while the slow-5 band was more sensitive in detecting additional results in the patients with acute BGIS compared with slow-4 and conventional bands. The sensitivity of slow-5 has been proposed in the study about poststroke depression [51]. Here, we provided the evidence from the perspective of acute BGIS patients. Our finding suggested that the combination of two measurements (ALFF and fALFF) could maximize the reliability of the study. In addition, our discovery of frequency-dependent results has had theoretical and clinical significance. The neural oscillations are not only the basic mechanism for coordinating activities during the normal operation of the brain but also the basic mechanism for stroke recovery, which has important reference value for the formulation of early rehabilitation treatment strategies for patients with BGIS.

Considering the prevalence of motor deficits in acute BGIS patients, the relationship between the altered brain regions detected by the ALFF and fALFF and motor function is worthy of attention. A study has revealed the increased connections between bilateral supramarginal gyrus and basal ganglia [52]. The supramarginal gyrus is a major component of the somatosensory association cortex which plays an important role in motor-related functions, such as integration of motor signals and various sensory data, transmission of integration information, and high level of cognitive motor control [53]. As for the medial frontal cortex, evidence from functional near infrared spectroscopy (fNIRS) proposed the importance of medial frontal cortex in motor response inhibition [54]. Also, Achala et al. [55] found that the medial frontal cortex was associated with manual motor response control.

Our results suggested that the right caudate was significantly associated with FMA score. The caudate belonged to the neostriatum and participated in the cortico-basal ganglia-thalamo-cortical loops and is involved in motor control, depression, and cognition [56, 57]. A study explored the relationship between lesion and FMA based on voxel-based lesion-symptom mapping. The result showed that declined motor outcome (walking capacity) tended to be affected by damage of the corona radiata, external capsule, and caudate [58]. One study examined structural connectivity changes of the motor execution network following therapy intervention after a stroke based on graph theory measures. The result showed that decreased degree centrality of caudate was associated with FMA. It should be noted that our interpretation is based on functional remolding of the cortico-basal ganglia-thalamo-cortical loops. Approaches targeting neural pathway may shed light on a better characterization and therapy innovation of stroke in the early-stage onset of stroke.

Several limitations should be acknowledged in the study. First, the sample size is relatively small in the present study. Larger sample size will be needed to confirm these results. Second, our research provided the different results in slow-5 and slow-4 bands measured by ALFF and fALFF in the acute BGIS patients. Studies which help to understand the underlying physiological mechanisms of altered ALFF and fALFF in each frequency band are also needed to be explored. Third, in order to eliminate the effects resulted from many times of stroke onset, we recruit merely first-onset acute patients with BGIS. The strict inclusion criteria lead to large gender differences between patient and HC groups, which may add some degree of selection bias.

## 5. Conclusion

In conclusion, this study showed the alterations in ALFF and fALFF in three frequency bands between patients with acute BGIS. The results reflected that the abnormal LFO amplitude might be related with different frequency bands and promoted our understanding of pathophysiological mechanism in acute BGIS.

## Figures and Tables

**Table 1:** Demographic and clinical characteristics of the participants.

	BGIS (*n* = 34)	HCs (*n* = 44)	*p* value
Age (years)	56.500 ± 10.999	55.340 ± 11.485	0.736
Gender (male/female)	25/9	19/25	0.007
Education (year)	11.500 ± 3.587		
NIHSS score	3.760 ± 2.463		
FMA score	74.910 ± 18.907		

BGIS: basal ganglia ischemic stroke; NIHSS: National Institutes of Health Stroke Scale; FMA: Fugl-Meyer Assessment.

**Table 2:** Brain regions showing ALFF and fALFF differences between groups.

Regions (AAL)	Brodmann area	Cluster size	Peak *t* value	MNI coordinate		
				*X*	*Y*	*Z*
ALFF						
Conventional band (0.01~ 0.08 Hz)						
SupraMarginal_R	40	111	-3.9736	36	-39	36
Slow-4 (0.027~ 0.073 Hz)						
SupraMarginal_R	40	117	-4.01	36	-39	36
fALFF						
Conventional band (0.01~ 0.08 Hz)						
Frontal_Mid_R	6	35	4.0499	42	6	60
Slow-4 (0.027~ 0.073 Hz)						
Frontal_Mid_R	6	27	3.7735	48	6	54
Slow-5 (0.01~ 0.027 Hz)						
Caudate_R	—	40	4.6062	18	6	15
Caudate_L	—	34	4.3024	-18	0	24

The clusters located in the cerebellum are not reported. AAL: automated anatomical labeling; MNI: Montreal Neurological Institute; SupraMarginal_R: right supramarginal gyrus; Frontal_Mid_R: right medial frontal gyrus; Caudate_R: right caudate; Caudate_L: left caudate; ALFF: amplitude of low frequency fluctuation; fALFF: fractional amplitude of low frequency fluctuation.

**Table 3:** The correlation results between brain regions and clinical measures.

Regions	Correlation values	
	NIHSS	FMA
ALFF		
Conventional frequency band (0.01-0.08 Hz)		
SupraMarginal_R	*r* = 0.044	*r* = −0.144
	*p* = 0.807	*p* = 0.415
Slow-4 frequency band (0.027-0.073 Hz)		
SupraMarginal_R	*r* = −0.010	*r* = −0.140
	*p* = 0.955	*p* = 0.429
fALFF		
Conventional frequency band (0.01-0.08 Hz)		
Frontal_Mid_R	*r* = 0.187	*r* = −0.114
	*p* = 0.289	*p* = 0.520
Slow-4 frequency band (0.027-0.073 Hz)		
Frontal_Mid_R	*r* = 0.070	*r* = 0.211
	*p* = 0.692	*p* = 0.231
Slow-5 frequency band (0.01-0.027 Hz)		
Caudate_R	*r* = 0.338	*r* = −0.363^∗^
	*p* = 0.051	*p* = 0.035
Caudate_L	*r* = 0.062	*r* = −0.024
	*p* = 0.729	*p* = 0.891

^∗^ *p* < 0.05. ALFF: amplitude of low-frequency fluctuations; fALFF: fractional amplitude of low-frequency fluctuations; SupraMarginal_R: right supramarginal gyrus; Frontal_Mid_R: right medial frontal gyrus; Caudate_R: right caudate; Caudate_L: left caudate; BGIS: basal ganglia ischemic stroke; NIHSS: National Institutes of Health Stroke Scale; FMA: Fugl-Meyer Assessment scale.

## Data Availability

The relevant raw data supporting the conclusions of this article will be made available upon request, without undue reservation.

## Supplementary Materials

Supplementary Materials Table S1: brain regions showing ALFF and fALFF differences between groups. Figure S1: a two-sample *t*-test was performed between BGIS patients and HCs. Figure S2: a two-sample *t*-test was performed between BGIS patients and HCs. Figure S3: lesion map for enrolled acute basal ganglia ischemic stroke.
