# Supplementary material for: Frequency-Specific Changes of Amplitude of Low-Frequency Fluctuations in Patients with Acute Basal Ganglia Ischemic Stroke
Source: Neural Plast. 2022 Jan 24;2022:4106131. doi: 10.1155/2022/4106131 (PMC8803449; doi:10.1155/2022/4106131)
Supplement: Supplementary Materials — Table S1: brain regions showing ALFF and fALFF differences between groups. Figure S1: a two-sample t-test was performed between BGIS patients and HCs. Figure S2: a two-sample t-test was performed between BGIS patients and HCs. Figure S3: lesion map for enrolled acute basal ganglia ischemic stroke. [file 4106131.f1.docx]

**Frequency-Specific Changes of Amplitude of Low‐Frequency Fluctuations in Patients with Acute Basal Ganglia Ischemic Stroke**

**Table S1.** Brain regions showing ALFF and fALFF differences between groups

| **Regions（AAL）** | **Brodmann Area** | **Cluster**  **Size** | **Peak**  ***t* Value** | **MNI Coordinate** | | |
| --- | --- | --- | --- | --- | --- | --- |
|  |  |  |  | **X** | **Y** | **Z** |
| **ALFF** | | | | | | |
| Slow-5(0.01-0.027Hz) | | | | | | |
| Caudate_R (aal) | - | 36 | 3.8886 | 18 | 6 | 12 |
| **fALFF** | | | | | | |
| Slow-5(0.01-0.027Hz) | | | | | | |
| Caudate_R (aal) | - | 29 | 4.1698 | 18 | 6 | 12 |
| Caudate_L (aal) | - | 29 | 3.8975 | -18 | 0 | 24 |

*AAL,* Automated anatomical labeling; *MNI,* Montreal Neurological Institute; *Caudate_R,* Right caudate; *Caudate_L,* Left caudate; *ALFF,* Amplitude of low frequency fluctuation; *fALFF,* Fractional amplitude of low frequency fluctuation.


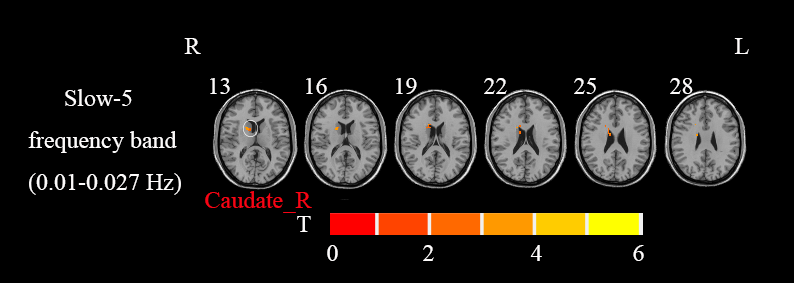


**Figure S1.** Two-sample *t*-test was performed between BGIS patients and HCs.

Abbreviations: *R*: right hemisphere; *L*: left hemisphere; *BGIS*: basal ganglia ischemic stroke; *HCs*: healthy controls; *Caudate_R,* Right caudate; *ALFF*: Amplitude of low frequency fluctuation.


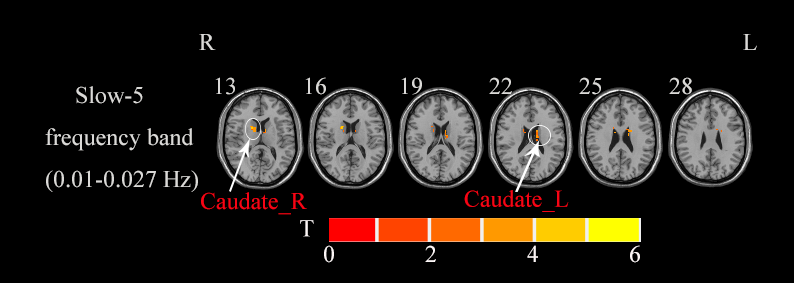


**Figure S2.** Two-sample *t*-test was performed between BGIS patients and HCs.

Abbreviations: *R*: right hemisphere; *L*: left hemisphere; *BGIS*: basal ganglia ischemic stroke; *HCs*: healthy controls; *Caudate_R,* Right caudate; *Caudate_L,* Left caudate; *fALFF*: Fractional amplitude of low frequency fluctuation


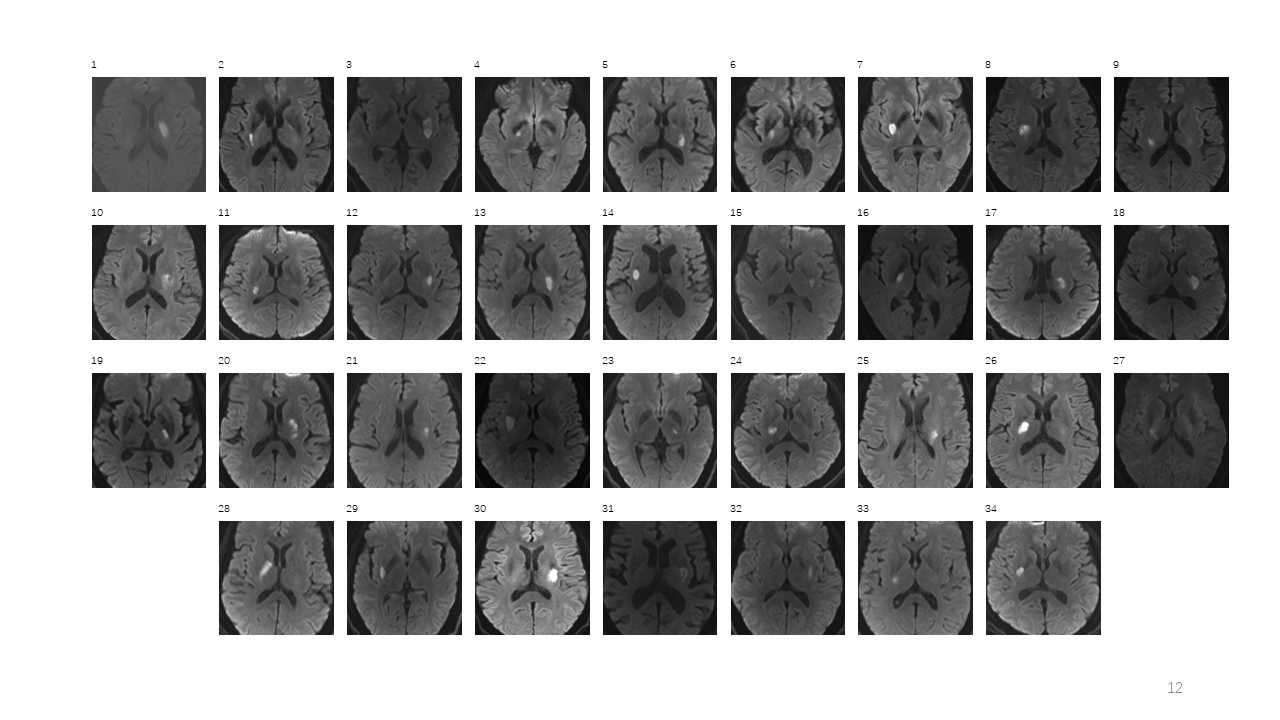


**Figure S3**. Lesion map for enrolled acute basal ganglia ischemic stroke
